# Nasal cartilage destruction associated to cutaneous histoplasmosis in AIDS

**DOI:** 10.1186/s12879-022-07351-0

**Published:** 2022-04-14

**Authors:** Luis Escalante, Jennyfer Granizo-Rubio, Victor Pinos-León, Sonia Tello, Astrid Maldonado, Iván Cherrez-Ojeda

**Affiliations:** 1 Unit of Dermatology, Central University of Ecuador, Quito, Ecuador; 2 Eugenio Espejo Hospital, Quito, Ecuador; 3 Derma Aid, Quito, Ecuador; 4 Department of Pathology, Hospital Axxis, Quito, Ecuador; 5 Universidad Espíritu Santo, Samborondón, Ecuador; 6 Respiralab Allergy and Respiratory Center, Samborondón, Ecuador

**Keywords:** Case report, Histoplasmosis, HIV, AIDS, Nasal cartilage destruction

## Abstract

**Background:**

Systemic histoplasmosis is a disease of high morbidity and mortality in immunocompromised patients. Patients with AIDS get the infection through inhalation of spores, triggering a primary lung infection with a subsequent hematogenous spread to multiple organs, including the skin. Tissue necrosis have been documented in cutaneous histoplasmosis with multiple clinical manifestations that mimic other diseases.

**Case presentation:**

We report the case of nasal cartilage destruction associated to cutaneous histoplasmosis in AIDS. A 24-year-old man, resident in Ecuadorian coast, with a history of HIV for 7 years without any treatment. In the last 3 months, he has been presenting a molluscum-like lesions on his nasal bridge with subsequent dissemination to the trunk and extremities. He was admitted to the emergency department for dyspnoea, cough, and malaise. Due to his respiratory failure, he was admitted to the intensive care unit (ICU) with mechanical ventilation. Physical examination reveals a crusted surface ulcer that involves the nose and cheeks, associated with erythematous papules, some with a crusted surface which are spread to the face, trunk, and upper limbs. The patient has a specific skin involvement with a butterfly-like ulcer appearance and destruction of the upper and lower lateral cartilage of the nose. At admission CD4 cell count was 11/mm^3^ with a HIV viral load of 322,908 copies. Mycological cultures identified Histoplasma capsulatum. A treatment with highly active antiretroviral therapy (HAART) was stablished, associated with liposomal amphotericin B at a dose of 3 mg/kg/day and itraconazole 200 mg twice a day for 12 months.

**Conclusions:**

Cutaneous histoplasmosis is a rare manifestation of pulmonary histoplasmosis in patients with AIDS. The cutaneous manifestations included papules, nodules, plaques, and ulcers. A histology examination is required to rule out other fungal or parasitic infections. Treatment includes highly active antiretroviral therapy (HAART), amphotericin B liposomal and itraconazole, the latest for at least 12 months.

## Background

Histoplasmosis is an endemic deep fungal infection caused by a dimorphic fungus Histoplasma capsulatum [1]. The disease is acquired through inhalation of the spores in endemic areas with large amount of spores [2]. In immunodeficient individuals, the clinical manifestations are variable. Patients with AIDS triggering a primary lung infection with a subsequent hematogenous spread to multiple organs, including the skin. Lower ratio of T:CD4 lymphocyte has been associated with cutaneous-systemic manifestations. In HIV patients, histoplasmosis induce atypical dysfunction of the tissue macrophages in the dermis with infiltration of inflammatory cells and cytokines with consequent tissue necrosis [3]. We report a young-male-patient with AIDS and systemic histoplasmosis with a complete destruction of the dorsal and lateral cartilage of the nose and a consequent flattening of the nose tip to the maxillary bone.

## Case presentation

A 24-year-old man seeking medical attention for slow growing molluscum-like lesions on his nasal bridge that spread to the trunk and extremities in the last 3 months. He has a history of HIV for 7 years without any treatment.

He was admitted to the emergency department for dyspnoea, cough, and malaise and due to his respiratory failure, he received mechanical ventilation.

**Fig. 1:**
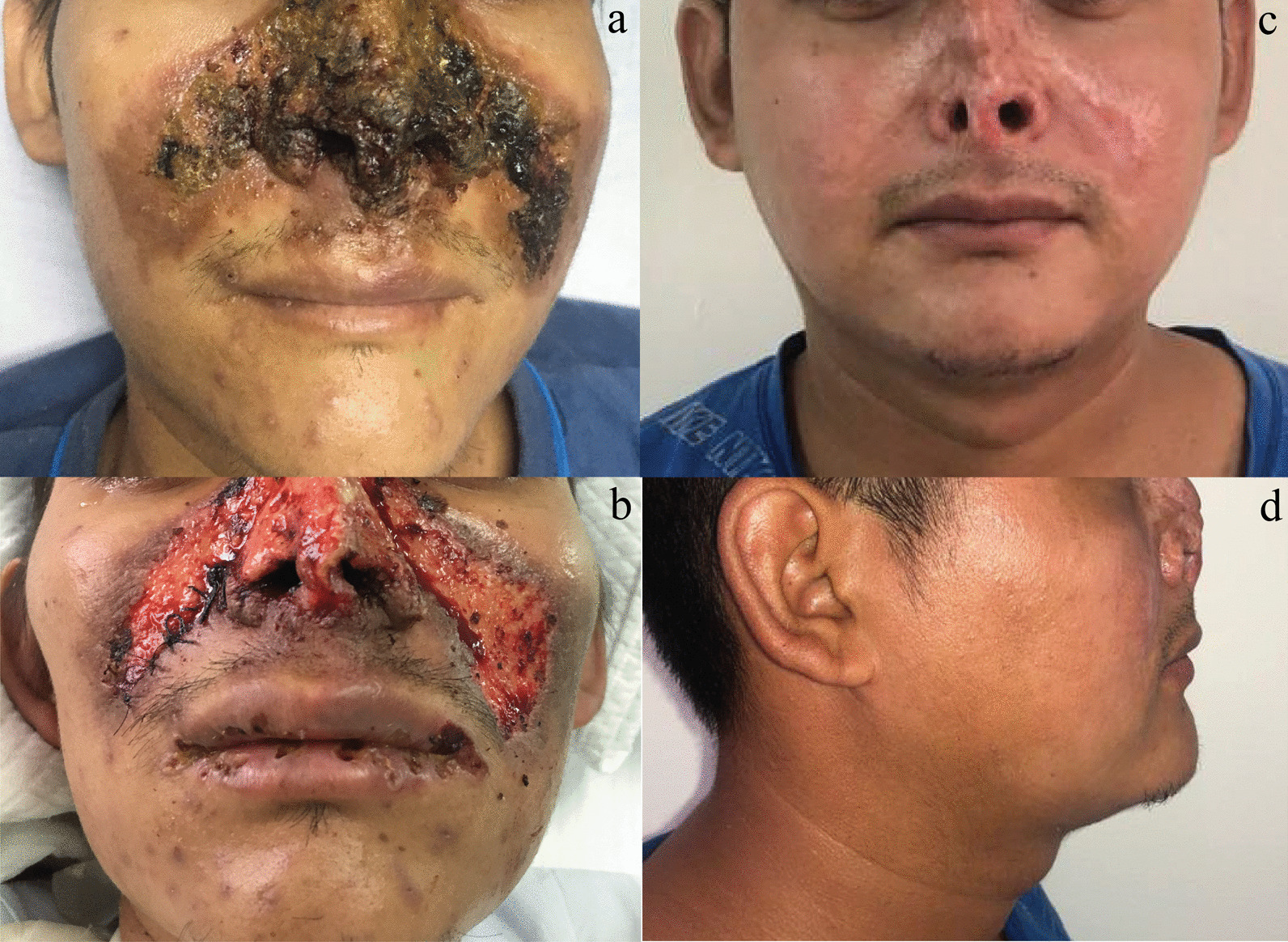
Clinical presentation of the case. **a** Localized central-facial ulcero-crusted plaque that involves the nose and cheeks with perilesional brownish erythema, compromising the nasal mucosa with a consequent reduction in the volume of the nasal pyramid. **b** Notice the flattening of the nasal tip due to the weakening of the nasal cartilage. **c**, **d** Result posttreatment with a complete destruction of the cartilage of the nose and consequent crushing on the nose tip. Source: Hospitalization

**Fig. 2:**
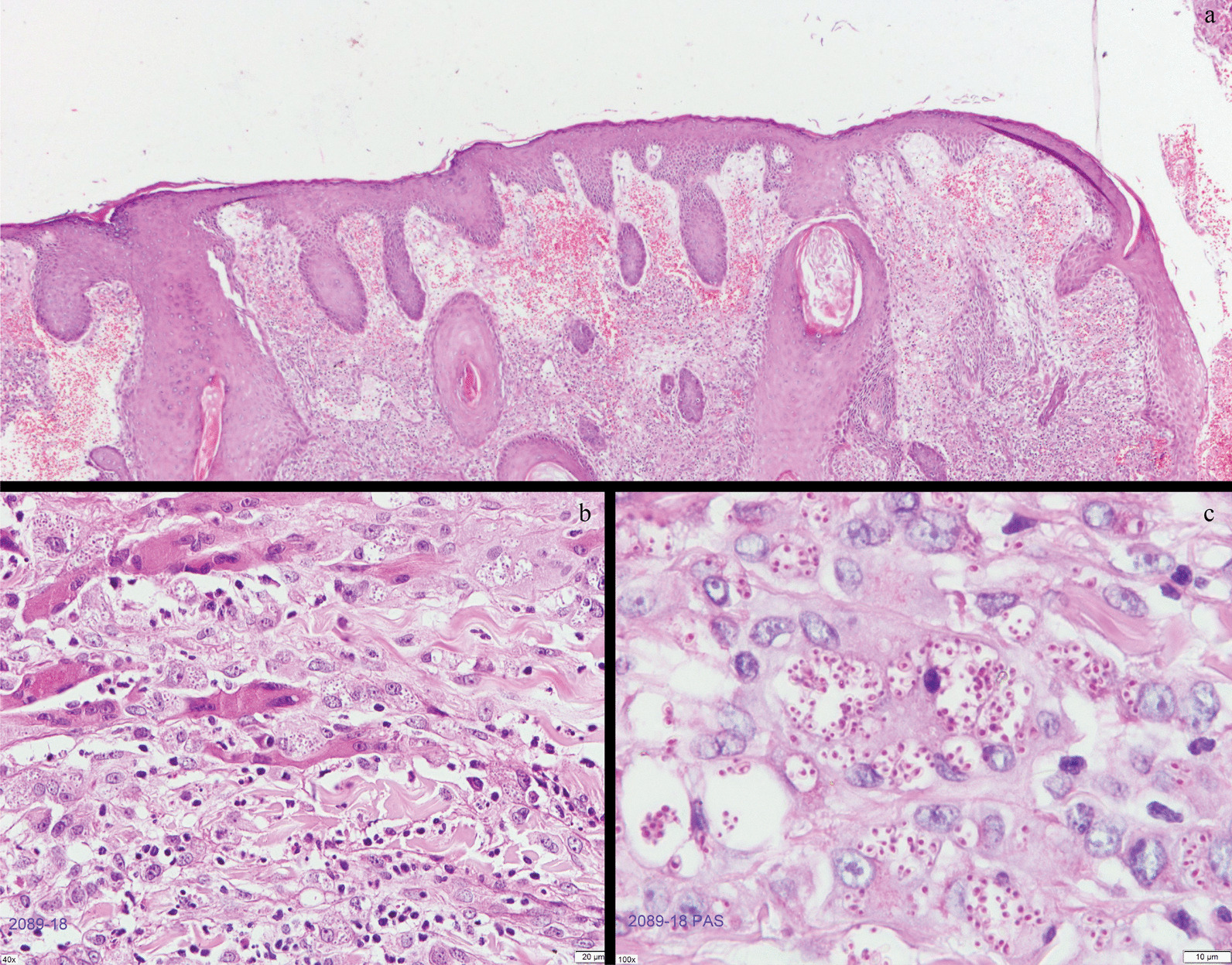
Histological analysis of the biopsy of the face. **a** Ulcerated epidermis alternating with areas of hyperkeratosis, papillomatosis, acanthosis, and irregular and pseudoepitheliomatous hyperplasia. **b** In the dermis, a diffuse lymphohistiocytic infiltrate is observed with multinucleated giant cells that contain many spores. **c** PAS staining. Source: Axxis Medical Laboratory

**Fig. 3:**
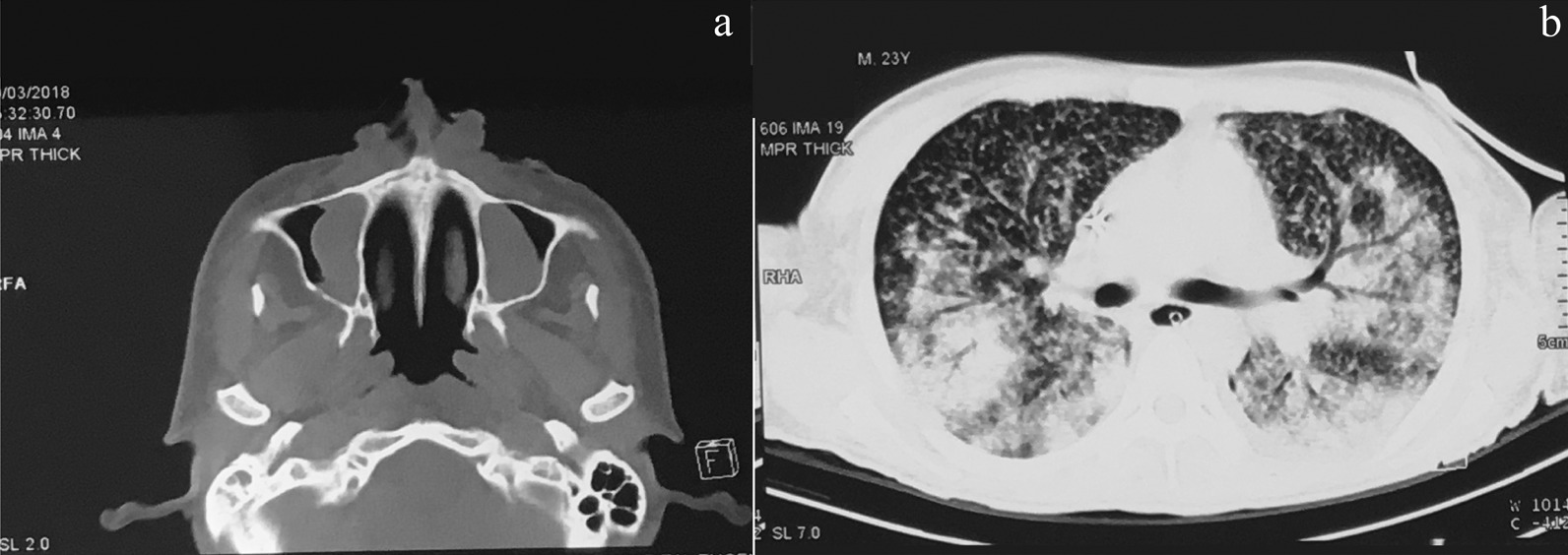
Imaging exploration. **a** Nasal CT scan showing a destruction of the dorsal, lateral, and alar cartilage of the nose. **b** Chest CT with bilateral pulmonary consolidations associated with reticular thickening and scattered pulmonary micronodules. Source: Hospitalization

Physical examination reveals a butterfly-like crusted ulcer appearance over the nose with a slow expanding surface until reach 5 cm on each side of the cheeks, associated with many erythematous papules some with a crusted surface which are spread to the face, trunk, and upper limbs (Fig. 1a–d). The cutaneous biopsy showed an ulcerated epidermis that alternates with areas of hyperkeratosis, papillomatosis, acanthosis, irregular and pseudoepitheliomatous hyperplasia. Oedema and vascular congestion were noted in the papillary dermis surrounded by a diffuse lymphohistocytic infiltrate with multinucleated giant cells that contain a large number of small spores of histoplasma-like fungi positive to with Periodic Acid-Schiff (PAS) staining. Necrosis, cellular debris, and neutrophil aggregates was abundant (Fig. 2a–c). Nasal CT scan showed a destruction of the dorsal, lateral, and alar cartilage of the nose. Lung CT scan showed bilateral pulmonary consolidations associated with reticular thickening and scattered pulmonary micronodules in more than 60% of the field (Fig. 3a, b). A final diagnosis of systemic histoplasmosis and nasal cartilage destruction was stablished with histoplasma PCR positive, and leishmania and tuberculosis PCR negative. A treatment with highly active antiretroviral therapy (HAART) was stablished, associated with liposomal amphotericin B at a dose of 3 mg/kg/day and itraconazole 200 mg twice a day for 12 months.

## Discussion and conclusion

Cutaneous-systemic histoplasmosis is an infection caused by a dimorphic fungus Histoplasma capsulatum, highly prevalent in Central and South America [4]. The infection is an indicator of AIDS with CD4 cell count less than 150 cells × mm^3^ [2, 5]. In accordance with that, our patient has 11 × mm^3^ with a viral load of 322,908 copies. The fungus can spread to many organs as liver, spleen, bone marrow, lymph nodes, gastrointestinal tract and central nervous system [6].

Oral histoplasmosis is a rare manifestation of systemic histoplasmosis and is hardly reported around the world. The spores of the fungus are present in areas with high humidity and abundant rainfall, especially in soils containing bird and bat faeces. In an immunocompetent patient, the infection usually is asymptomatic. Primary infection is acquired through the inhalation of microconidia that once, in the tissues grow as dimorphic yeasts, reaching a morbidity and mortality of up to 39% in immunocompetent patients [7].

Despite the existence of defensive innate immunity mechanisms such as leukocytes, macrophages, NK cells, antigen-presenting cells and complement, this first line of defence is incapable by itself to control the fungus. The infection affects the lungs, and then by hematogenous spread it can reach other organs. Cutaneous manifestations were developed in up to 25% in men with HIV. The skin and mucosal involvement is highly frequent [8, 9].

Cutaneous histoplasmosis include papules, nodules, plaques, pustules, acneiform eruptions, umbilicated papules resembling molluscum contagiosum lesions and ulcers, the latter mimic other fungal or parasitic infections [10].

Nasomaxillar histoplasmosis is extremely rare; and palate, gingiva, and tongue are the most frequently locations reported of disseminated histoplasmosis that compromise central facial area. We present a patient with complete destruction of the nasal cartilage due to histoplasmosis with a butterfly shape. Compromise of the nasal and oral mucosa has been reported, although with a lower prevalence. The diagnosis is made by histology and periodic acid Schiff (PAS) stain, which is considered the gold standard. Although the culture is highly specific, it has several limitations including a variable sensitivity in HIV patients between 75% and 85–95% [1, 3, 11–13].

The destruction of nasal cartilage is the result of hematogenous dissemination of the fungus spores, triggering a cascade of pro-inflammatory cytokines and the cytogenetic effects generated by tissue macrophages, lymphocytes and neutrophiles that progressively destroy the fine and delicate nasal cartilage [14].

Differential diagnoses include drug rash, eosinophilic folliculitis, prurigo secondary to HIV, scabies, psoriasis, penicilliosis, and other bacterial and fungal infections. Due to its necrotic appearance mucormycosis must be considered first, however the prevalence is low in Ecuador. In our endemic area, cutaneous ulcer and nasal destruction could be a misleading Leishmaniasis, especially due to the similar clinical and histologic manifestations between the intracell amastigotes and histoplasma. Also, primary cutaneous tuberculosis should be considered in endemic areas due to his pulmonary and verrucous cutaneous compromission. In the context of central facial ulcer-necrotic disease, clinician should rule out, NK/T cell-lymphoma, squamous-cell carcinoma, Wegener’s granulomatosis, and sarcoidosis [15–18].

In literature, only 19 cases of central facial involvement due to Histoplasma have been reported, 13 of them had AIDS with CD4 cell counts < 150/mm^3^, and 3 died because of delay or non-adherence to treatment [12]. Treatment include with liposomal amphotericin B, itraconazole and HAART in HIV positive patients [12, 19–28].

Our patient received liposomal amphotericin B 3 mg/kg/day for two weeks, followed by itraconazole 200 mg orally twice a day for one year. Antifungal therapy has been determined to continue until serum and urine anti-histoplasma antigens were < 2.0 ng/mL [17, 18]. After 1 year of treatment, Itraconazole 200 mg/day should be continued until blood cultures negative for Histoplasma, antigenuria or antigenemia < 2 ng/mL in patients with more than 150 CD4/mL on antiretroviral therapy for more than 6 months (29).

There are many clinical presentations of this infection which usually start as primary lung histoplasmosis and can compromise central facial area.

There are previous reports of nasal septum perforation that does not include the entire nasal pyramid and butterfly-like appearance. To the best of our knowledge, this is the first reported case of a complete destruction of the nasal cartilage by histoplasma in a butterfly shape.

A prompt response to treatment is expected to prevent fatal outcomes, especially when clinician has to considerer many other etiologies that mimic this presentation.

## Data Availability

Not applicable.

## Abbreviations

AIDS: Acquired immune deficiency syndrome
HIV: Human immunodeficiency virus
PAS: Periodic Acid-Schiff
HAART: Highly active antiretroviral therapy
ICU: Intensive care unit

## References

[1] Arango-Bustamante K, Restrepo A, Cano LE, De Bedout C, Tobón AM, González A (2013). Diagnostic value of culture and serological tests in the diagnosis of histoplasmosis in HIV and non-HIV Colombian patients. Am J Trop Med Hyg.

[2] Joseph Wheat L, Connolly-Stringfield PA, Baker RL, Curfman MF, Eads ME, Israel KS (1990). Disseminated histoplasmosis in the acquired immune deficiency syndrome: clinical findings, diagnosis and treatment, and review of the literature. Medicine (United States)..

[3] de Oliveira RD, de Alencar RRFR, Antonio BVR, Leal PC, dos Santos Franco E, dos Santos LM. Discrete cutaneous lesions in a critically ill patient treated only for AIDS and miliary tuberculosis: a case report of disseminated histoplasmosis. Dermatol Online J. 2019;25(8). 31553862

[4] Sánchez-Saldaña L, Galarza C, Cortéz FF (2010). Infecciones micóticas sistémicas o profundas: histoplasmosis. Dermatol Peru.

[5] da Silva Ferreira B, de Araújo Filho JA, Pereira NM, de Miranda Godoy L, Lamounier BB, Nunes ED (2017). Disseminated histoplasmosis in aids patients: an urban disease. Experience in a metropolis in the middle east of Brazil. Infez Med.

[6] Soza GM, Patel M, Readinger A, Ryan C (2016). Disseminated cutaneous histoplasmosis in newly diagnosed Hiv. Baylor Univ Med Cent Proc.

[7] Sirait SP, Bramono K, Hermanto N (2017). Correlation of CD4 counts with clinical and histopathological findings in disseminated histoplasmosis: a 10-year retrospective study. Int J Dermatol.

[8] Pérez-Lazo G, Maquera-Afaray J, Mejia CR, Castillo R (2017). Histoplasmosis diseminada e infección por VIH: Serie de casos en un hospital peruano. Rev Chil Infectol.

[9] Orsi AT, Nogueira L, Chrusciak-Talhari A, Santos M, Ferreira de LCL, Talhari S (2011). Histoplasmosis and AIDS co-infection. An Bras Dermatol.

[10] Vasudevan B, Ashish B, Amitabh S, Mohanty A (2010). Primary cutaneous histoplasmosis in a HIV-positive individual. J Glob Infect Dis.

[11] Daneri AGL, Arechavala A, Iovannitti CA, Mujica MT. Artículo original histoplasmosis diseminada en pacientes HIV/SIDA. BUENOS AIRES, 2009–2014 Resultados Se estudiaron las historias clínicas de los 171 casos de Materiales y métodos. 2016;332–7. 27959839

[12] Lehur AC, Zielinski M, Pluvy J, Grégoire V, Diamantis S, Bleibtreu A (2017). Case of disseminated histoplasmosis in a HIV-infected patient revealed by nasal involvement with maxillary osteolysis. BMC Infect Dis.

[13] Cáceres DH, Gómez BL, Restrepo Á, Tobón ÁM (2012). Histoplasmosis y sida: factores de riesgo clínicos y de laboratorio asociados al pronóstico de la enfermedad. Infection.

[14] Oikawa F, Carvalho D, Matsuda NM, Yamada AT (2010). Histoplasmosis in the nasal septum without pulmonary involvement in a patient with acquired immunodeficiency syndrome: case report and literature review. Sao Paulo Med J.

[15] Özer M, Özsurekçi Y, Cengiz AB, Özçelik U, Yalçin E, Gököz Ö (2016). Primary Nasal Tuberculosis in a 10-Year-Old Girl. Can J Infect Dis Med Microbiol.

[16] Neetu B, Piyush P, Arava S, Gupta S. Histoplasmosis mimicking non-Hodgkin lymphoma in a 40-year-old man with AIDS. Int J STD AIDS 2017; 28:312–4. Available from: journals.sagepub.com/home/std 10.1177/0956462416665942 27535728

[17] Myint T, Anderson AM, Sanchez A, Farabi A, Hage C, Baddley JW (2014). Histoplasmosis in patients with human immunodeficiency virus/acquired immunodeficiency syndrome (HIV/AIDS): multicenter study of outcomes and factors associated with relapse. Medicine (United States).

[18] Mangalore RP, Moso MA, Cronin K, Young K, McMahon JH (2018). Treatment of disseminated histoplasmosis in advanced HIV using itraconazole with increased bioavailability. Int J STD AIDS.

[19] Schlaen A, Ingolotti M, Couto C, Jacob N, Pineda G, Saravia M (2015). Endogenous Histoplasma capsulatum endophthalmitis in an immunocompetent patient. Eur J Ophthalmol.

[20] Rizzi MD, Batra PS, Prayson R, Citardi MJ (2006). Nasal histoplasmosis. Otolaryngol Head Neck Surg.

[21] Butt AA, Carreon J (1997). Histoplasma capsulatum sinusitis. J Clin Microbiol.

[22] Alves MD, Pinheiro L, Manica D, Fogliatto LM, Fraga C, Goldani LZ (2011). Histoplasma capsulatum sinusitis: case report and review. Mycopathologia.

[23] Elansari R, Abada R, Rouadi S, Roubal M, Mahtar M (2016). Histoplasma capsulatum sinusitis: possible way of revelation to the disseminated form of histoplasmosis in HIV patients: case report and literature review. Int J Surg Case Rep.

[24] Felix F, Gomes GA, Pinto PCL, Arruda AM, Marques MDPC, Tomita S (2006). Nasal histoplasmosis in the acquired immunodeficiency syndrome. J Laryngol Otol.

[25] Souza Filho FJ, Lopes M, Almeida OP, Scully C (1995). Mucocutaneous histoplasmosis in AIDS. Br J Dermatol.

[26] Jaimes A, Muvdi S, Alvarado Z, Rodríguez G (2013). Perforation of the nasal septum as the first sign of histoplasmosis associated with AIDS and review of published literature. Mycopathologia.

[27] Machado AA, Coelho ICB, Roselino AMF, Trad ES, de Figueiredo JFC, Martinez R (1991). Histoplasmosis in individuals with acquired immunodeficiency syndrome (AIDS): report of six cases with cutaneous-mucosal involvement. Mycopathologia.

[28] Manzini M, Lavinsky-Wolff M (2012). Nasal histoplasmosis without lung involvement in an immunocompromised patient. Braz J Otorhinolaryngol.

[29] Panel on Opportunistic Infections in Adults T. Guidelines for the Prevention and Treatment of Opportunistic Infections in Adults and Adolescents with HIV How to Cite the Adult and Adolescent Opportunistic Infection Guidelines: Panel on Opportunistic Infections in Adults and Adolescents with HIV. Guidelines for the prevention and treatment of opportunistic infections in adults and adolescents [Internet]. Available from: http://aidsinfo.nih.gov/contentfiles/lvguidelines/adult_oi.pdf. Accessed 19675369

